# BMI Status of Children with Celiac Disease Has Changed in the Last Decades: A 30-Year Retrospective Study

**DOI:** 10.3390/nu16162729

**Published:** 2024-08-16

**Authors:** Alice Monzani, Silvia Marcolin, Federico Medina, Kevin Valentino, Ivana Rabbone

**Affiliations:** 1 Division of Pediatrics, Department of Health Sciences, University of Piemonte Orientale, Via Solaroli 17, 28100 Novara, Italy; 20011805@studenti.uniupo.it (F.M.); kevin.valentino.93@gmail.com (K.V.); ivana.rabbone@uniupo.it (I.R.); 2 Italian Celiac Association, Piedmont Section, 10136 Turin, Italy; s.marcolin@aicpiemonte.it

**Keywords:** celiac disease, children, body mass index, clinical presentation

## Abstract

The presenting pattern of celiac disease (CD) at diagnosis in children has changed over time, with a reduction of malabsorption-related phenotypes and an increase in regular or even excessive growth patterns. We retrospectively reviewed the body mass index (BMI) distribution of all patients with a new diagnosis of CD made in a Pediatric Gastroenterology Outpatient Clinic in 1990–2011, compared to those diagnosed in 2012–2022, according to their clinical and serological characteristics. The 1990–2011 and 2012–2022 cohorts included 250 (M:F = 90:160, mean age 7.3 ± 6.1 years) and 243 children (M:F = 81:162, mean age 7.1 ± 3.7 years, NS), respectively. The prevalence of underweight (UW) was higher in the 1990–2011 cohort (61/250, 24.4% in 1990–2011 vs. 31/243, 12.7% in 2012–2022, *p* = 0.0001), whereas that of overweight (OW) and obese (OB) subjects was significantly higher in 2012–2022 (10/250, 4% in 1990–2011 vs. 24/243, 9.9% in 2012–2022, *p* = 0.012, and 1/250, 0.4% in 1990–2011 vs. 8/243, 3.3% in 2012–2022, *p* = 0.018, respectively). In both cohorts, gastrointestinal symptoms were more frequent in OW/OB than in UW children (6/11, 54.5% vs. 5/61, 8.2% in 1990–2011, *p* < 0.0001, and 24/32, 75% vs. 10/31, 32.3%, *p* < 0.0001 in 2012–2022), and the extent of anti-transglutaminase antibody increase was similar in OW/OB and UW subjects. The prevalence of children with a normal or even high BMI at CD diagnosis has increased in the past three decades; therefore, CD should be suspected regardless of BMI status.

## 1. Introduction

Over the decades, the presenting pattern of celiac disease (CD) at diagnosis in children has changed, both for the presenting symptoms and clinical characteristics. If the classical presentation in the past was that of a young child with failure to thrive, diarrhea, and malabsorption, we are now facing more and more frequently with older children, often presenting with extra-intestinal symptoms or even asymptomatic, and with a regular or even excessive weight at diagnosis [1,2,3,4,5]. With specific regard to the body mass index (BMI) status, an increase in overweight and obese subjects, along with a reduction in underweight ones at diagnosis, has been reported [6], even if the prevalence of overweight/obesity among subjects with CD is still lower than that of the general population [7]. In adults, up to 32% of patients with celiac disease presented with overweight or obesity [8], and overweight/obese subjects are reported to be mainly male, with a higher age and significantly lower anti-tissue transglutaminase antibody levels at diagnosis compared to the normal weight patient cohort [9]. Furthermore, no association between the severity of histological findings and BMI has been reported [10]. In pediatric age, a recent systematic review reported a prevalence of overweight or obesity in school-age children (6–17 years) affected by CD ranging between 3.5% and 20% [11]. Conversely, in a cohort of children with overweight/obesity screened for CD, a diagnosis of CD was reported in 4% [12].

We aimed to retrospectively review all the new diagnoses made in a Pediatric Gastroenterology Unit over the past 30 years to assess how the clinical presentation has changed, with particular regard to BMI status distribution.

## 2. Materials and Methods

This was a single-center retrospective study conducted by recruiting all children and adolescents aged 0–18 years who received a new diagnosis of celiac disease at the Pediatric Gastroenterology Outpatient Clinic of Maggiore Della Carità University Hospital in Novara, Piedmont, Italy, in the period 1990–2011, compared to those diagnosed in 2012–2022. The two cohorts were divided by choosing 2012—the year of the introduction of ESPGHAN guidelines introducing the possibility of a biopsy-sparing approach [13]—as the breakpoint. 

For each subject, the following data were collected:

- Demographic characteristics: sex, age at diagnosis.
- Anthropometric data: height was measured with a standard laboratory stadiometer (Holtain, Wales, UK) to the nearest 0.5 cm during maximal expiration. Weight was measured to the nearest 100 g in light indoor clothing with a spring scale tested daily for accuracy and calibrated against a set of standard weights (Salus, Inc., Milan, Italy). All measurements taken by the same operator were repeated twice, and if they differed more than 0.5 cm in height and 100 g in weight, a third measure was taken. The average of the two closest measures was used. BMI (body mass index) was calculated as weight/height^2^ (kg/m^2^) and categorized according to BMI cut-offs of the International Obesity Task Force [14] into underweight (UW, BMI < 3° percentile), normal weight (NW, BMI 3–75° percentile), overweight (OW, BMI 75–95° percentile), and obesity (OB, BMI > 95° percentile).
- Clinical characteristics: for each subject, the main presenting symptoms were collected and categorized as recurrent abdominal pain, diarrhea, constipation, failure to thrive, anemia, fatigue, headache, dermatitis herpetiformis, and other symptoms or no symptoms referred. The presence of one or more first-degree relatives with CD, as well as the concomitant diagnosis of other CD-related diseases (autoimmune thyroiditis, type 1 diabetes, Down syndrome, Turner syndrome), was collected. The modality of CD diagnosis, with or without biopsy, was also reported.
- Serological data: maximum anti-transglutaminase IgA antibody (TGA-A) titer at diagnosis was collected. As not all the patients performed TGA-A measurements in the same laboratory, TGA-A values were expressed as the number of times the upper limit of normal (× ULN) for each laboratory.

Comparisons of demographic, clinical, and serological data were made between underweight and overweight/obese subjects in the historical and recent cohorts.

The study was conducted in adherence to the regulations established by the local Ethics Committee (EC), the Declaration of Helsinki, and Good Clinical Practice guidelines. Informed written consent was obtained from all subjects’ parents and from the patients, where appropriate, and the local Ethics Committee approved the study protocol (CE015/2024).

### Statistical Analysis

Descriptive statistics were performed to summarize the main characteristics of the subjects included in the study. Categorical variables were reported as absolute frequencies and percentages, while numerical variables were reported as mean and standard deviation. Two-tailed chi-square or Fisher’s exact test was used to evaluate differences in prevalence, as appropriate. The Mann–Whitney U test was used to compare continuous variables. A 5% level of significance was adopted in this work. The statistical analysis was performed using SPSS version 28.0 (Chicago, IL, USA).

## 3. Results

**Figure 1:**
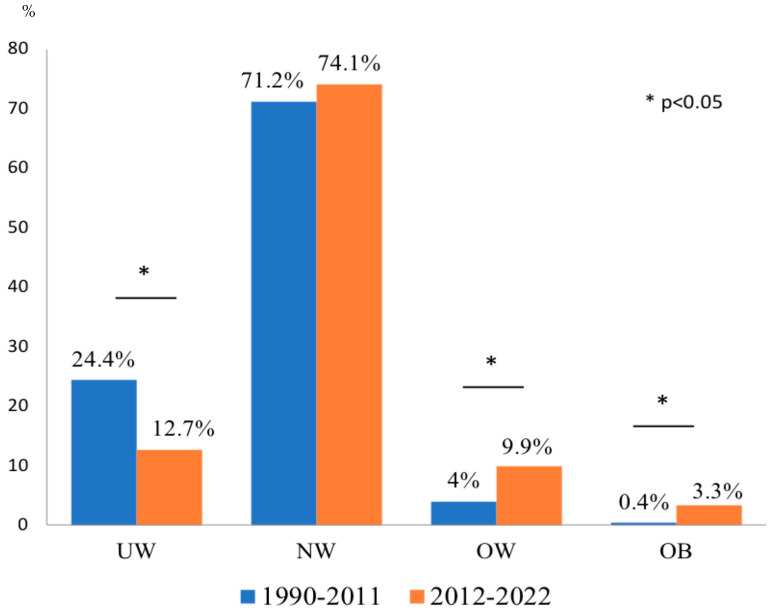
BMI status at CD diagnosis in 1990–2011 vs. 2012–2022. UW: underweight, NW: normal weight, OW: overweight; OB: obesity.

The 1990–2011 cohort included 250 subjects (M:F = 90:160, mean age 7.3 ± 6.1 years) and the 2012–2022 cohort 243 subjects (M:F = 81:162, mean age 7.1 ± 3.7 years, *p* = 0.54). The distribution of patients according to BMI status in the cohorts is shown in Figure 1. The prevalence of UW was significantly higher in the 1990–2011 cohort (61/250, 24.4% in 1990–2011 vs. 31/243, 12.7% in 2012–2022, *p* = 0.0001). The prevalence of NW subjects was similar in the two cohorts (178/250, 71.2% in 1990–2011 vs. 180/243, 74.1% in 2012–2022, *p* = 0.47). The prevalence of OW subjects was significantly higher in 2012–2022 than in 1990–2011 (24/243, 9.9% vs. 10/250, 4%, *p* = 0.012), as was the prevalence of OB subjects (8/243, 3.3% vs. 1/250, 0.4%, *p* = 0.018).

Comparing OW/OB with UW subjects, in the 2012–2022 cohort, OW/OB children were older than UW (8.43 ± 3.9 vs. 6.35 ± 2.9 years, *p* = 0.03) (Table 1). In the 1990–2011 cohort, all children performed endoscopy for CD diagnosis, as per current guidelines, whereas in the 2012–2022 cohort, a biopsy-sparing approach was reported in 142/243 new diagnoses (58.4%). The frequency of biopsy-sparing diagnosis was similar in UW and OW/OB children (Table 1). Both in the 1990–2011 and the 2012–2022 cohorts, the extent of the TGA-A increase was similar in OW/OB and UW children (Table 1).

Clinical characteristics at diagnosis according to BMI status are reported in Table 2. Regarding presenting symptoms, gastrointestinal symptoms (abdominal pain, diarrhea, and constipation) taken together were significantly more frequent in OW/OB than in UW children both in the 1990–2011 cohort (6/11, 54.5% vs. 5/61, 8.2%, *p* < 0.0001) and the 2012–2022 cohort (24/32, 75% vs. 10/31, 32.3%, *p* < 0.0001). Among gastrointestinal symptoms, in 1990–2011, OW/OB children more frequently presented with constipation, whereas in 2012–2022, abdominal pain was the most reported. In both cohorts, failure to thrive was the most frequent presenting symptom among UW subjects (79.7% in 1990–2011 and 48.3% in 2012–2022). The distribution of extra-intestinal symptoms was similar between OW/OB and UW children in both cohorts, as was the prevalence of asymptomatic children.

The prevalence of a positive family history of CD was not significantly different across different BMI classes in both cohorts, even if in 2012–2022 it was nearly double in OW/OB than in UW subjects (25% vs. 12.9%). The frequency of CD-related conditions such as autoimmune thyroiditis, type 1 diabetes, and Down or Turner syndrome was higher in OW/OB children than in UW children in 1990–2011, but similar in 2012–2022.

## 4. Discussion

In our cohort, we observed a substantial shift in the phenotype of newly diagnosed children with CD over time: the prevalence of underweight decreased by half, that of overweight more than doubled, and that of obesity became three times higher.

Several hypotheses have been formulated to explain the association between celiac disease and overweight/obesity, foremost among them being the “compensatory” hypothesis [15]. According to this theory, the distal portion of the intestine not affected by mucosal damage and atrophy undergoes morphological changes, including increased villi height, elongation of crypts, and an increase in the number of enterocytes, to compensate for malabsorption in the atrophic duodenum—jejunum, similarly to what occurs in the residual intestine after surgical resection. This can increase absorption in the functionally preserved intestinal tract, and if the process leads to overcompensation, it can result in a greater energy intake than the subject’s needs, thereby increasing the risk of overweight/obesity. The intestinal surface undergoing this process tends to increase with the patient’s age, explaining the age-related distribution of symptoms at the diagnosis of celiac disease: patients under 2 years of age often present with classical manifestations, including malabsorption, unlike older children and adolescents who generally report atypical symptoms, including increased BMI. Indeed, in our series, we showed that patients with overweight/obesity are older than underweight ones, also suggesting that a normal or even excess weight might determine a diagnostic delay.

Another factor potentially responsible for the increased number of celiac patients with high BMI at diagnosis is the worldwide increase in the prevalence of overweight/obesity observed [16]. In Italy, however, the global prevalence of overweight/obesity in children has recently slightly decreased over time: according to a European study, the prevalence of overweight/obesity in Italian schoolchildren decreased by 6.9% from 2007/2208 to 2015/2017 [17], and according to a national registry, overweight and obesity decreased from 23.6% and 12.3% in 2008 to 19% and 9.8% in 2023, respectively [18]. Conversely, in our cohort of children with CD, we observed an increase in overweight/obesity prevalence, respectively, from 4.4% to 13.2%, in about 30 years. This may suggest a rise over time in clinicians’ awareness of CD without signs of malabsorption and, therefore, a greater propension to thinking about CD even in children with normal or high BMI status.

To further possibly disentangle which factor—the “compensatory” hypothesis or the global increase in overweight/obesity among children—plays a major role in the explanation of the increased BMI at CD diagnosis, it would be useful to assess how the BMI status possibly changes during a gluten-free diet (GFD). Assuming that the histological recovery of the duodenal mucosa after a variable period of strict adherence to the GFD would also revert the hyper-absorbent pattern of the distal intestine, we would expect a BMI normalization in a quite large number of subjects that had a weight excess at diagnosis. A concordant evolution was reported by Reilly et al. in a cohort of US children with CD, in which 75% of patients with an elevated BMI at diagnosis decreased their BMI z-scores after a GFD, normalizing it in 44% of cases [10]. However, conflicting results have been reported. Venkatasubramani et al. showed that BMI decreased in 57% and increased in 28% of children that were obese at diagnosis [19], and Valletta et al. reported that the percentage of overweight subjects almost doubled after at least 12 months of GFD [20]. Large studies and long-term follow-ups are needed to assess the real impact of the GFD on BMI status in CD children, taking into account many variables, such as GFD compliance, symptom resolution, dietary counseling, and adequacy of nutritional behavior. Undoubtedly, a follow-up by an expert dietitian would be advisable to tailor the nutritional intervention and optimize GFD adherence, with the aim of reverting potential abnormal BMI status at CD diagnosis, possibly correcting both the deficiency and the excess.

To further characterize the children presenting with overweight or obesity at CD diagnosis, we analyzed their presenting symptoms, finding that gastrointestinal symptoms are the main complaint reported more frequently than underweight subjects, similar to what was described by Reilly [10] and Venkatasubramani [19]. Concerning serological data at diagnosis, we did not find differences in the extent of TTG-A elevation between underweight and overweight/obese subjects in both cohorts, similar to what was reported by Singh et al. in Indian adolescents and adults with CD [21]. In previous reports, controversial data emerged in children with CD: Capriati et al. showed that overweight/obese subjects had significantly lower levels of tTG antibodies than their normal-weight counterparts [9], whereas Shahraki et al. reported that overweight/obese and normal-weight subjects had significantly higher levels of tTG antibodies than underweight ones [22].

The higher prevalence of CD-related disorders and the trend toward an increased prevalence of positive family history for CD we found in overweight/obese subjects suggest that children with a high BMI status are more likely to be screened for CD only in the presence of a predisposing condition than their counterparts with a normal or low BMI.

## 5. Conclusions

In conclusion, it emerges that BMI cannot be considered a reliable tool for selecting patients with probable CD. Still, any patient with suggestive symptoms, signs, or predisposing condition should be screened for CD, regardless of BMI status, to possibly avoid diagnostic delays due to falsely reassuring normal or excess growth. From this perspective, it would be of great interest to assess how the BMI status distribution at CD diagnosis would change after the forthcoming implementation of national mass screening for CD in the Italian pediatric population [23].

## Figures and Tables

**Table 1:** Demographic and diagnostic data at diagnosis of CD in the 1990–2011 and 2012–2022 cohorts, according to BMI status (UW vs. OW/OB). UW: underweight; OW: overweight; OB: obesity.

	1990–2011 Cohort			2012–2022 Cohort		
	UW n = 61	OW/OB n = 11	*p*	UW n = 31	OW/OB n = 32	*p*
Age, years (mean, SD)	6.5 ± 5.0	11.1 ± 6.6	0.07	6.35 ± 2.9	8.43 ± 3.9	0.03
Sex, males (n, %)	25 (41%)	5 (45.4%)	0.48	12 (38.7%)	19 (59.4%)	0.10
Biopsy-sparing diagnosis (n, %)	0	0	1	16 (51.6%)	16 (50%)	0.90
TGA-A × ULN (mean, SD)	14.5 ± 7.1	7.8 ± 8.7	0.11	156.7 ± 457.0	152.8 ± 451.6	0.30

**Table 2:** Clinical and anamnestic data of children at diagnosis of CD in the 1990–2011 and 2012–2022 cohorts, according to BMI status (UW vs. OW/OB). UW: underweight; OW: overweight; OB: obesity.

	1990–2011 Cohort			2012–2022 Cohort		
	UW n = 61	OW/OB n = 11	*p*	UW n = 31	OW/OB n = 32	*p*
Abdominal pain	3 (4.9%)	2 (18.2%)	0.11	8 (25.8%)	20 (62.5%)	0.003
Diarrhea	2 (3.3%)	2 (18.2%)	0.04	2 (6.4%)	2 (6.2%)	0.97
Constipation	0 (0%)	3 (27.3%)	0.003	0 (0%)	2 (6.2%)	0.49
Failure to thrive	48 (79.7%)	0 (0%)	<0.0001	15 (48.3%)	0 (0%)	<0.0001
Anemia	0 (0%)	0 (0%)	1	0 (0%)	2 (6.2%)	0.49
Fatigue	1 (1.8%)	2 (18.2%)	0.06	3 (9.6%)	1 (3.1%)	0.35
Headache	0 (0%)	0 (0%)	1	0 (0%)	0 (0%)	1
Dermatitis herpetiformis	1 (1.8%)	0 (0%)	1	0 (0%)	2 (6.2%)	0.49
Other symptoms	5 (8.2%)	0 (0%)	1	1 (3.5%)	0 (0%)	0.49
No symptoms	1 (1.8%)	2 (18.2%)	0.06	2 (6.4%)	3 (9.4%)	1
Positive family history of CD	1 (1.6%)	0 (0%)	1	4 (12.9%)	8 (25%)	0.34
CD-related disease(s)	0 (0%)	2 (18.2%)	0.02	9 (29%)	7 (21.9%)	0.57

## Data Availability

The raw data supporting the conclusions of this article will be made available by the authors upon request.
